# Can telomere shortening be the main indicator of non-viable fetus elimination?

**DOI:** 10.1186/s13039-018-0361-9

**Published:** 2018-01-25

**Authors:** Nataliya Huleyuk, Iryna Tkach, Danuta Zastavna, Miroslaw Tyrka

**Affiliations:** 1 Institute of Hereditary Pathology, NAMS of Ukraine, Lysenko Str. 31a, Lviv, 79008 Ukraine; 2 Department of Biotechnology and Bioinformatics, Faculty of Chemistry, Rzeszow University of Technology, Al. Powstańców Warszawy 6, 35-959 Rzeszow, Poland

**Keywords:** Relative telomere length, Spontaneous abortions, Aneuploidy, Euploidy

## Abstract

**Background:**

Telomeres are transcriptionally inactive genomic areas, which, if shortened, are associated with pathological processes, unsuccessful fertilization, aging, and death. Telomere dysfunction has also been linked to chromosomal rearrangements and genomic instability. The role of telomeres in postnatal life has been extensively studied and discussed both in physiological as well as in pathological processes. However, the role of telomere length in prenatal development is still poorly understood, and mainly concerns the preimplantation stage. The aim of this study was to estimate relative telomere length in spontaneously eliminated human embryos between 5th and 12th week of gestation.

**Results:**

Relative telomere length was measured from total genomic DNA using a real-time polymerase chain reaction approach. In this study, we examined relative telomere length in 80 spontaneously eliminated embryos and in 25 embryos eliminated due to induced abortions. Relative telomere length in spontaneous abortions was significantly lower (*P* = 0.000001) compared to the induced abortions. Spontaneous abortions with aneuploid anomalies (monosomy X, trisomy 21, trisomy 16 and triploidy) were characterized by shorter telomeres, compared to spontaneous abortions, subgroup with euploid (46,XN) karyotype.

**Conclusion:**

Spontaneously lost pregnancies are characterized by shortened telomeres, especially in embryos with aneuploidies. We hypothesize that the shortening of telomeres is involved in the processes leading to spontaneous abortions.

## Background

Telomeres are transcriptionally inactive areas of the human genome, involved in maintenance of genomic integrity. Telomere DNA consists of 10–15 kb long hexamer iterations (TTAGGG)n [1, 2], which end with a 3′-single-helixed area, creating a D-loop [3, 4]. Formation of telomeric loop takes place with support of the shelterin complex, which consists of 6 proteins (TRF1, TRF2, TIN2, RAP1, TPP1 and POT1). The main function of this complex is to prevent degradation of telomeres [5]. During each cellular division, telomeric DNA is shortened by 50–200 bp [6–8]. As soon as the length of telomeres becomes critically low, the cells stop dividing and enter apoptosis [9, 10]. Thus, telomeres are believed to be the so-called cellular clock, which controls division and cellular death. Moreover, telomere dysfunction is linked to chromosomal rearrangements, genomic instability, tumorigenesis and cellular senescence [11, 12].

Telomere length (TL) is proposed to be an indicator of biological age of an organism, and a significant correlation between age and TL has been shown [13]. Role of telomeres during postnatal development was intensely studied and discussed both for normal and pathological conditions. In particular, telomere length is decreased in diabetes mellitus [14], cardiovascular disease [15], liver disorders [16, 17], cancer [18], and severe premature aging phenotypes [19]. Decreased TL has also been linked to adiposity [20, 21], low social and economic status [22], chronic emotional stress [23], smoking [24], increased mortality [25] and others.

However, the role of telomere length in prenatal human development remains mostly unknown, both under pathological as well as under physiological conditions, and the available data is contradictory [26–29]. Few recent studies mainly focused on preimplantation stages of human embryonic development available due to preimplantation diagnostic procedures [30–33]. According to the recent data, aneuploid human polar bodies possess significantly shorter telomeres than euploid polar bodies from sibling oocytes, although, at the blastocyst stage, telomeres did not differ in euploid and aneuploid embryos [30, 31]. It is also likely that the chance of successful in vitro fertilization decreases along with the decrease in TL in oocytes. In particular, it has been shown by Keefe et al. [32, 33] that oocytes from women who did not conceive after in vitro fertilization had shorter telomeres compared to those who did. In addition, oocytes from cycles that produced fragmented embryos also had shorter telomeres.

In summary, shortened telomeres were already associated with pathological processes, unsuccessful fertilization, aging, and death. In this regard, here we estimated the relative telomere length in spontaneously aborted human embryos at 5–12 weeks of gestation (w.o.g). In addition, the results of this pioneering study were interpreted taking into account the presence of chromosomal anomalies.

## Methods

In this work, we have studied relative telomere lengths (RTL) in 80 chorionic villi samples (CVS) from spontaneously eliminated product of conception at 5–12 weeks of gestational age (spontaneous abortions - SA) and from 25 induced abortions (IA) due to personal reasons at the same term of gestation. All studied pregnancies occurred naturally. Maternal age ranged from 20 to 34 years.

### Collection of chorionic villi samples

A total of 105 chorionic villi samples (CVS) at 5–12 weeks of gestation were obtained from pregnant women, 80 of them from spontaneous abortions with the following selected karyotype:

- 46,XX or 46,XY – 32 samples;
- 69,XXN – 13 samples;
- trisomy 16–13 samples;
- trisomy 21–10 samples;
- monosomy X – 12 samples;

and 25 samples from induced abortions with euploid karyotype 46,XN (46,XX or 46,XY).

All CVS samples were tested for absence of maternal deciduae by histological analysis. Samples were washed twice with PBS and stored at − 20 °C until DNA extraction.

### DNA extraction

Genomic DNA was extracted using salting-out or phenol/chloroform method, and dissolved in Tris-EDTA pH 8.0. DNA concentration and purity was assessed using Qubit® 2.0 Fluorometer (Thermo Fisher Scientific). All DNA samples were stored at − 20 °C until telomere length analysis.

### RTL measurement by real-time PCR

Relative telomere length was measured from total genomic DNA using a real-time PCR assay [34]. PCR reactions were performed in the Eco Real-Time PCR System (Illumina, Inc), using 1X GoTaq® qPCR Master Mix (Promega) and specific primers (Table 1) in recommended concentrations [34].

**Table 1:** List of specific primers used for determination of telomere length

Primer name	Sequence (5′-3′)
Tel 1	GGTTTTTGAGGGTGAGGGTGAGGGTGAGGGTGAGGGT
Tel 2	TCCCGACTATCCCTATCCCTATCCCTATCCCTATCCCTA
36B4u	CAGCAAGTGGGAAGGTGTAATCC
36B4d	CCCATTCTATCATCAACGGGTACAA

Two reaction mixes of PCR reagents were prepared, one for amplification of telomere repeats (T), the other for gene *36B4* (S). Each PCR reaction contained 12 μl of the reaction mix (GoTaq® qPCR Master Mix and primers) and 7 μl (14 ng/aliquot) DNA. Tel1 and tel2 primers for telomeres were added to the final concentrations 270 nM and 900 nM, respectively. The final *36B4* gene primer concentrations were: 36B4u - 300 nM and 36B4d - 500 nM. For each sample in whom T/S ratio was measured, tree identical aliquots of DNA were added to plate T (telomere) and another tree aliquots were added to the same well positions in plate SCG (single copy gene). Reference DNA was included into each PCR run.

**Fig. 1:**
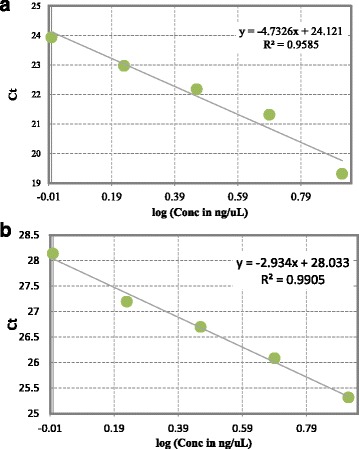
Standard curves used for calculation of relative DNA concentrations of telomeres (**a**) and 36B4 (**b**)

The reference DNA for measurement standardization was obtained from whole blood of two healthy individuals (male and female). Standard curves for telomere length and for the single copy gene were generated by performing serial dilutions (dilution factor 1.68) from a reference DNA sample to produce concentrations of DNA ranging from 0.63 to 5 ng/μl in two parallel reactions – one for the telomeric sequences and the other for the reference gene (36B4) (Fig. 1). The data obtained from reference DNA measurement were used to setup calibration curves needed to estimate reaction efficiency and the average RTL. The relative ratio of telomere repeats copy number to *36B4* gene copy number (T/S ratio) in experimental samples were compared to the reference DNA sample. Telomere length was expressed as a relative T/S ratio, based on the calculation of the ΔCt [Ct_(telomere)_/Ct_(single gene)_] value, which was normalized to the average T/S ratio of the reference sample [2^-(ΔCt(sample)- ΔCt(control)^ = 2^-ΔΔCt^].

Illumina Eco Software v4.1.11.2 was used to generate curves for the telomere signal (Т) or the single copy gene signal (S) and to determine quantity of DNA for our research.

The coefficient of variation (standard deviation/mean) was calculated to be 0.89% for within plate measurements and 0.77% for measurement between plates.

### Statistical analysis

The data acquired from RLT measurement was normally distributed (Shapiro-Wilk test, *p* = 0.00001). Statistical differences between means were tested with ANOVA and t-test using Statistica 12 (StatSoft, Inc., USA).

## Results

The average RTL in the combined group was 0.60 and was characterized by inter-individual variations within the range of 0.03 to 2.94. In the IA group, the average RTL was 1.17 with inter-individual variations ranging from 0.14 to 2.94. In the main SA investigated group, the mean of RTL was 0.43 with inter-individual variations ranging from 0.03 to 2.40 (Table 2).

**Table 2:** Relative telomere length ratios in spontaneous and artificial abortions overall and within each group

Overall		Spontaneous abortions		Artificial abortions	
Mean (max-min)	N	Mean (max-min)	n	Mean (max-min)	n
0.60 (0.03–2.94)	105	0.43 (0.03–2.40)	80	1.17 (0.14–2.94)	25

n- number of samples

**Fig. 2:**
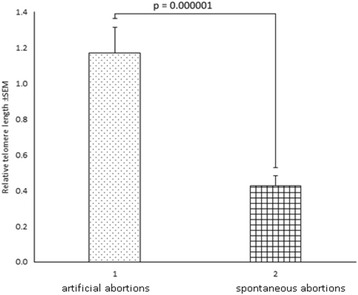
Relative telomere length in chorionic villus samples. 1 – artificial abortions; 2 – spontaneous abortions

Statistical analysis showed that RTL were significantly lower in SA group compared to IA group (SA: 0.43 ± 0.06 vs IA: 1.17 ± 0.14, *P* = 0.000001) (Fig. 2).

The SA group was further divided into subgroups depending on the karyotype. Molecular cytogenetic (interphase mFISH with centromeric probe panels for chromosomes 13, 21, 14, 22, 15, 16, 17, 18, X and Y) and cytogenetic studies showed the following results in this group: 32 cases with euploid karyotype (46,XX or 46,XY) and 48 with aneuploid karyotype (autosomal trisomy 21 or 16, triploidy, monosomy X). Variability of the mean RTL for SA with or without chromosome number abnormalities are shown in Table 3.

**Table 3:** Relative telomere length ratios in spontaneous abortions with euploid or aneuploid karyotype

Overall		Euploid karyotype		Aneuploid karyotype	
Mean (max-min)	n	Mean (max-min)	n	Mean (max-min)	n
0.43 (0.03–2.40)	80	0.64 (0.03–2.40)	32	0.29 (0.07–1.66)	48

n- number of samples

As indicated in Table 3, the aneuploid SA was characterized by shorter telomeres in comparison to the euploid SA with interindividual variations from 0.07 to 1.66 and from 0.03 to 2.40, respectively.

**Fig. 3:**
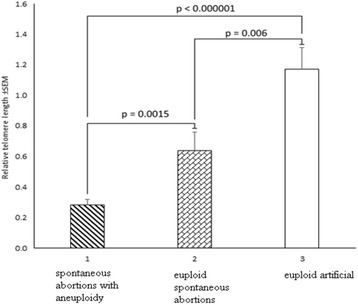
Relative telomere length in artificial and spontaneous abortions with or without aneuploidy. 1 – spontaneous abortions with aneuploidy; 2 – euploid spontaneous abortions; 3 – euploid artificial abortions

Statistical analysis showed a highly significant difference in RTL between SA with aneuploid and SA with euploid karyotype (aneuploid: 0.29 ± 0.03 vs euploid: 0.64 ± 0.12, *P* = 0.0015) (Fig. 3).

**Fig. 4:**
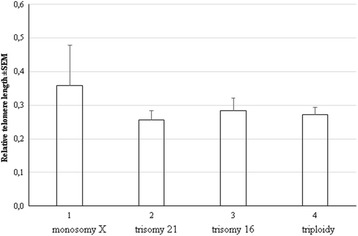
Relative telomere length in spontaneous abortions with different aneuploidies. 1 – monosomy X; 2 – trisomy 21; 3 – trisomy 16; 4 - triploidy

Further, we compared RTL in SA with different types of aneuploidy - trisomy chromosome 21, trisomy chromosome 16, triploidy and monosomy X (Table 4). The results showed no significant variation in RTL length among the various aneuploid groups (monosomy X: 0.36 ± 0.12; trisomy 21: 0.26 ± 0.03; trisomy 16: 0.28 ± 0.04; and triploidy: 0.27 ± 0.02, *P* > 0.05) (Fig. 4). Consequently, the telomere length does not depend on the kind of aneuploidy in CVS of spontaneously lost pregnancies.

**Table 4:** Relative telomere length ratios in spontaneous abortions with different form of aneuploidy

Monosomy X		Trisomy 21		Trisomy 16		Triploidy	
Mean (max-min)	n	Mean (max-min)	n	Mean (max-min)	n	Mean (max-min)	n
0.36(0.08–1.66)	12	0.26(0.11–0.35)	10	0.28(0.07–0.49)	13	0.27(0.11–0.37)	13

n- number of samples

It should be noted that aneuploidy was not detected in IA group. In fact, euploid karyotype (46,XX or 46,XY) was found in all 25 samples. Therefore, we compared relative telomere length between euploid SAs and IAs and observed a highly significant difference in RTL (0.64 ± 0.12 and 1.17 ± 0.14 respectively, *P* = 0.006). These results indicate that the shortening of telomeres seems to play a role in the early human intrauterine interruption of further growth and development (Fig. 3).

**Fig. 5:**
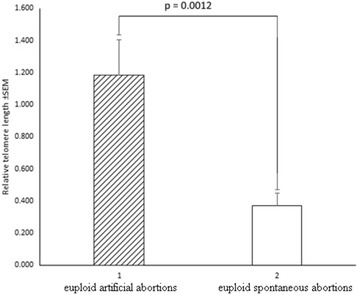
Relative telomere length in chorionic villus samples with karyotype 46,XY. 1 – euploid artificial abortions; 2 – euploid spontaneous abortions

**Fig. 6:**
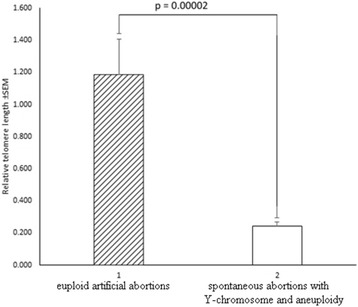
Relative telomere length in artificial chorionic villus samples with karyotype 46,XY and spontaneous abortions with Y-chromosome and aneuploidy. 1 – euploid artificial abortions; 2 – spontaneous abortions with Y-chromosome and aneuploidy

In addition, aware of the possibility of contamination of embryonic material with maternal cells, we compared the groups only with male karyotypes (Table 5, Fig. 5, Fig. 6). A total of 48 male embryos were examined: 14 - IA, 15 - euploid SA, 19 - aneuploid SA. Apparently, a significant difference is maintained when comparing the XY-euploid IA to XY-euploid SA embryos (*P* = 0.0012) and even increased (*P* = 0.00002) when comparing IA to aneuploid SA, further strengthening our hypothesis.

**Table 5:** Relative telomere length ratios in chorionic villus samples with Y-chromosome

Spontaneous abortions with euploid karyotype		Spontaneous abortions with aneuploid karyotype		Artificial abortions	
Mean (max-min)	n	Mean (max-min)	n	Mean (max-min)	n
0.37(0.30–1.13)	15	0.24(0.07–0.49)	19	1.86(0.14–2.94)	14

n- number of samples

## Discussion

Based on our and data obtained by others [35–46], it can be stated that telomere length is characterized by interindividual variability both in prenatal and in the postnatal human development. To date, very few studies focus on telomere length in the early stages of human prenatal development. Therefore, our study provides a unique example of studying RTL in early (5–12 w.o.g.) prenatal development and evaluate it according to anamnesis (SA and IA) and in association with aneuploid (autosomal trisomy, monosomy X and triploidy) and euploid karyotype.

Our results show, with a high degree of reliability (*P* = 0.000001), that spontaneously lost pregnancies are characterized by short telomeres in comparison to induced abortions. Therefore, we report a strong correlation between telomere length and the viability of embryos. In particular chronic stress in pregnant women is associated with short telomeres in posterity [41, 43, 44]. In addition, a lower maternal folate concentration in early pregnancy is associated with shorter telomeres in the newborn [45]. Genomic instability is commonly found in newborns with short telomeres, which increases the risk of cancer and age-related diseases [46]. Our data shows significantly shorter RTL in spontaneously eliminated embryos with aneuploid (with trisomy of chromosome 21 or 16, triploidy and monosomy X) karyotype, regardless of the type of aneuploidy. Similar results were shown [30, 31] for aneuploid oocytes and embryos at the cleavage stage, although the telomere length was aligned at the blastocyst stage. The results of studies of telomere length in newborns with trisomy 21 (Down syndrome) are conflicting - from the claim of shortening [47] or lack of a likely difference in telomere length [48] to a probable elongation of telomeres in newborns with trisomy 21 versus newborns without chromosomal anomalies [49]. Similarly, ambiguous results are obtained in adults with monosomy X (Turner syndrome) [50], which indicate rather that there is no difference in the length of telomeres in Turner syndrome cells compared to individuals without chromosomal abnormalities.

## Conclusion

Spontaneously lost pregnancies are characterized by shortened telomeres, especially in embryos with aneuploidies.. We hypothesize that the shortening of telomeres is involved in the complex elimination machinery leading to early embryo death.

## Abbreviations

CVS: Chorionic villi samples
IA: Induced abortions
RTL: Relative telomere length
RT-PCR: Real-time polymerase chain reaction
S: Single copy gene
SA: Spontaneous abortions
T: Telomere repeat
TL: Telomere length
w.o.g: Weeks of gestation

## References

[1] Blackburn EH (1991). Structure and function of telomeres. Nature.

[2] Cairney CJ, Keith WN (2008). Telomerase redefined: integrated regulation of hTR and hTERT for telomere maintenance and telomerase activity. Biochimie.

[3] Griffith JD, Comeau L, Rosenfield S, Stansel RM, Bianchi A, Moss H, de Lange T (1999). Mammalian telomeres end in a large duplex loop. Cell.

[4] De Lange T (2002). Protection of mammalian telomeres. Oncogene.

[5] De Lange T (2005). Shelterin: the protein complex that shapes and safeguards human telomeres. Genes Dev.

[6] Zhao Y, Sfeir AJ, Zou Y, Buseman CM, Chow TT, Shay JW, Wright WE (2009). Telomere extension occurs at most chromosome ends and is uncoupled from fill-in in human cancer cells. Cell.

[7] Watson JD (1972). Origin of concatemeric T7 DNA. Nat New Biol.

[8] Sfeir AJ, Chai W, Shay JW, Wright WE (2005). Telomere-end processing the terminal nucleotides of human chromosomes. Mol Cell.

[9] Hayflick L (1965). The limited in vitro lifetime of human diploid cell strains. Exp Cell Res.

[10] Arkus N (2005). A mathematical model of cellular apoptosis and senescence through the dynamics of telomere loss. J Theor Biol.

[11] Mai S, Garini Y (2006). The significance of telomeric aggregates in the interphase nuclei of tumor cells. J Cell Biochem.

[12] Bernadotte A, Mikhelson VM, Spivak IM (2016). Markers of cellular senescence. Telomere shortening as a marker of cellular senescence. Aging (Albany NY).

[13] Hunt SC, Chen W, Gardner JP, Kimura M, Srinivasan SR, Eckfeldt JH, Berenson GS, Aviv A (2008). Leukocyte telomeres are longer in African Americans than in whites: the National Heart, Lung, and Blood Institute family heart study and the Bogalusa heart study. Aging Cell.

[14] Murillo-Ortiz B, Albarran-Tamayo F, Arenas-Aranda D, Benitez-Bribiesca L, Malacara-Hernandez J, Martinez-Garza S, Hernández-González M, Solorio S, Garay-Sevilla ME, Mora-Villalpando C (2012). Telomere length and type 2 diabetes in males, a premature aging syndrome. Aging Male.

[15] Fitzpatrick AL, Kronmal RA, Kimura M, Gardner JP, Psaty BM, Jenny NS, Tracy RP, Hardikar S, Aviv A (2011). Leukocyte telomere length and mortality in the cardiovascular health study. J Gerontol A Biol Sci Med Sci.

[16] Hartmann D, Srivastava U, Thaler M, Kleinhans KN, N’Kontchou G, Scheffold A, Bauer K, Kratzer RF, Kloos N, Katz SF, Song Z, Begus-Nahrmann Y, Kleger A, von Figura G, Strnad P, Lechel A, Günes C, Potthoff A, Deterding K, Wedemeyer H, Ju Z, Song G, Xiao F, Gillen S, Schrezenmeier H, Mertens T, Ziol M, Friess H, Jarek M, Manns MP, Beaugrand M, Rudolph KL (2011). Telomerase gene mutations are associated with cirrhosis formation. Hepatology.

[17] Calado RT, Brudno J, Mehta P, Kovacs JJ, Wu C, Zago MA, Chanock SJ, Boyer TD, Young NS (2011). Constitutional telomerase mutations are genetic risk factors for cirrhosis. Hepatology.

[18] Martinez-Delgado B, Yanowsky K, Inglada-Perez L, Domingo S, Urioste M, Osorio A, Benitez J (2011). Genetic anticipation is associated with telomere shortening in hereditary breast cancer. PLoS Genet.

[19] Calado RT, Young NS (2009). Telomere diseases. N Engl J Med.

[20] Nordfjall K, Eliasson M, Stegmayr B, Lundin S, Roos G, Nilsson PM (2008). Increased abdominal obesity, adverse psychosocial factors and shorter telomere length in subjects reporting early ageing; the MONICA northern Sweden study. Scand J Public Health.

[21] Njajou OT, Cawthon RM, Blackburn EH, Harris TB, Li R, Sanders JL, Newman AB, Nalls M, Cummings SR, Hsueh W-C (2012). Shorter telomeres are associated with obesity and weight gain in the elderly. Int J Obes.

[22] Cherkas LF, Hunkin JL, Kato BS, Richards JB, Gardner JP, Surdulescu GL, Kimura M, Lu X, Spector TD, Aviv A (2008). The association between physical activity in leisure time and leukocyte telomere length. Arch Intern Med.

[23] Epel ES, Blackburn EH, Lin F, Dhabhar FS, Adler NE, Morrow JD, Cawthon RM (2004). Accelerated telomere shortening in response to life stress. Proc Natl Acad Sci U S A.

[24] Valdes AM, Andrew T, Gardner JP, Kimura M, Oelsner E, Cherkas LF, Aviv A, Spector TD (2005). Obesity, cigarette smoking, and telomere length in women. Lancet.

[25] Cawthon RM, Smith KR, O’Brien E, Sivatchenko A, Kerber RA (2003). Association between telomere length in blood and mortality in people aged 60 years or older. Lancet.

[26] Ferrari F, Facchinetti F, Saade G, Menon R (2016). Placental telomere shortening in stillbirth: a sign of premature senescence?. J Matern Fetal Neonatal Med.

[27] Menon R, Yu J, Basanta-Henry P, Brou L, Berga SL, Fortunato SJ, Taylo RN (2012). Short fetal leukocyte telomere length and preterm prelabor rupture of the membranes. PLoS One.

[28] Biron-Shental T, Sukenik-Halevy R, Sharon Y, Goldberg-Bittman L, Kidron D, Fejgin MD, Amiel A (2010). Short telomeres may play a role in placental dysfunction in preeclampsia and intrauterine growth restriction. Am J Obstet Gynecol.

[29] Davy P, Nagata M, Bullard P, Fogelson NS, Allsopp R (2009). Fetal growth restriction is associated with accelerated telomere shortening and increased expression of cell senescence markers in the placenta. Placenta.

[30] Treff NR, Su J, Taylor D, Scott RT (2011). Telomere DNA deficiency is associated with development of human embryonic aneuploid. PLoS Genet.

[31] Mania A, Mantzouratou A, Delhanty JD, Baio G, Serhal P, Sengupta SB (2014). Telomere length in human blastocysts. Reprod BioMed Online.

[32] Keefe DL, Franco S, Liu L, Trimarchi J, Cao B, Weitzen S, Agarwal S, Blasco MA (2005). Telomere length predicts embryo fragmentation after in vitro fertilization in women—toward a telomere theory of reproductive aging in women. Am J Obstet Gynecol.

[33] Keefe DL, Liu L, Marquard K (2007). Telomeres and aging-related meiotic dysfunction in women. Cell Mol Life Sci.

[34] Cawthon RМ (2002). Telomere measurement by quantitative PCR. Nucleic Acids Res.

[35] Holmes DK, Bellantuono I, Walkinshaw SA, Alfirevic Z, Johnston TA, Subhedar NV, Chittick R, Swindell R, Wynn RF (2009). Telomere length dynamics differ in foetal and early post-natal human leukocytes in a longitudinal study. Biogerontology.

[36] Cheng G, Kong F, Luan Y, Sun C, Wang J, Zhang L, Jiang B, Qi T, Zhao J, Zheng C, Xu D. Differential shortening rate of telomere length in the development of human fetus. Biochem Biophys Res Commun. 2013;442(1–2):112-5. 10.1016/j.bbrc.2013.11.022. 10.1016/j.bbrc.2013.11.022 24246679

[37] Youngren K, Jeanclos E, Aviv H, Kimura M, Stock J, Hanna M, Skurnick J, Bardeguez A, Aviv A (1998). Synchrony in telomere length of the human fetus. Hum Genet.

[38] Turner S, Wong HP, Rai J, Hartshorne GM (2010). Telomere lengths in human oocytes, cleavage stage embryos and blastocysts. Mol Hum Reprod.

[39] Okuda K, Bardeguez A, Gardner JP, Rodriguez P, Ganesh V, Kimura M, Skurnick J, Awad G, Aviv A (2002). Telomere length in the newborn. Pediatr Res.

[40] Akkad A, Hastings R, Konje JC, Bell SC, Thurston H, Williams B (2006). Telomere length in small for gestational age babies. BJOG Int J Obstet Gynaecol.

[41] Entringer S, Epel ES, Lin J, Buss C, Shahbaba B, Blackburn EH, Simhan HN, Wadhwa PD (2013). Maternal psychosocial stress during pregnancy is associated with newborn leukocyte telomere length. Am J Obstet Gynecol.

[42] Armanios M, Blackburn EH (2012). The telomere syndromes. Nat Rev Genet.

[43] Marchetto NM, Glynn RA, Ferry ML, Ostojic M, Wolff SM, Yao R, Haussmann MF (2016). Prenatal stress and newborn telomere length. Am J Obstet Gynecol.

[44] Entringer S, Epel ES, Kumsta R, Lin J, Hellhammer DH, Blackburn EH, Wüst S, Wadhwa PD (2011). Stress exposure in intrauterine life is associated with shorter telomere length in young adulthood. Proc Natl Acad Sci U S A.

[45] Entringer S, Epel ES, Lin J, Blackburn EH, Buss C, Shahbaba B, Gillen DL, Venkataramanan R, Simhan HN, Wadhwa PD (2015). Maternal Folate concentration in early pregnancy and newborn telomere length. Ann Nutr Metab.

[46] Moreno-Palomo J, Creus A, Marcos R, Hernández A (2014). Genomic instability in newborn with short telomeres. PLoS One.

[47] Wenger SL, Hansroth J, Shackelford AL (2014). Decreased telomere length in metaphase and interphase cells from newborns with trisomy 21. Gene.

[48] Nakamura K, Ishikawa N, Izumiyama N, Aida J, Kuroiwa M, Hiraishi N, Fujiwara M, Nakao A, Kawakami T, Poon SS, Matsuura M, Sawabe M, Arai T, Takubo K (2014). Telomere lengths at birth in trisomies 18 and 21 measured by Q-FISH. Gene.

[49] Bhaumik P, Bhattacharya M, Ghosh P, Ghosh S, Kumar DS (2017). Telomere length analysis in down syndrome birth. Mech Ageing Dev.

[50] Kveiborg M, Gravholt CH, Kassem M (2001). Evidence of a normal mean telomere fragment length in patients with Ullrich-turner syndrome. Eur J Hum Genet.

